# Preparation, physicochemical properties, and immunomodulatory activity of glycoproteins from *Syngnathoides biaculeatus*

**DOI:** 10.1007/s13659-025-00551-6

**Published:** 2025-12-01

**Authors:** Xuewei Xia, Jieling Lin, Lihong Yang, Youhong Li, Wenshen Lin, Qingqing Wang, Jun Liu, Riming Huang

**Affiliations:** 1 Guangdong Provincial Key Laboratory of Food Quality and Safety, College of Food Science, South China Agricultural University, Guangzhou, 510642 China; 2 Guangdong Eco-Engineering Polytechnic, Guangzhou, 510520 China; 3 Guangzhou University of Chinese Medicine, Guangzhou, 510006 China; 4 Laboratory of Pathogenic Biology, Guangdong Medical University, Zhanjiang, 524023 China

**Keywords:** *Syngnathoides biaculeatus*, Glycoprotein, Physicochemical properties, Immunomodulatory activity

## Abstract

*Syngnathoides biaculeatus*, a potential functional food from marine sources, was found to enhance nonspecific immunity, but its functional ingredients have rarely been reported. Therefore, this study focused on the preparation and physicochemical properties of its water-soluble natural ingredients with their immunomodulatory activities and potential mechanisms. Firstly, we optimized the extraction method of glycoproteins from *S. biaculeatus* and prepared the crude glycoprotein SYB, from which the fraction glycoprotein SYB-1 was further purified. The carbohydrates of SYB-1 were 8.46% comprising mannose, glucose, and galactose, with an average molecular weight of 9.423 kDa. Amino acid analysis demonstrated that its major amino acids are glycine, glutamic acid, aspartic acid, and proline, with a total amino acid content of 88.81%. Furthermore, SYB-1 could significantly increase the cell viability of macrophages, and promote the release of NO, TNF-*α*, and IL-6. Metabolomics revealed that it was associated with arachidonic acid metabolism. The CYP450 enzyme family members and PTGS2 may be key targets for the regulatory role. These results suggested that the glycoprotein of *S. biaculeatus* may be an attractive functional food supplement from natural sources for immunocompromised populations.

**Graphical Abstract:**

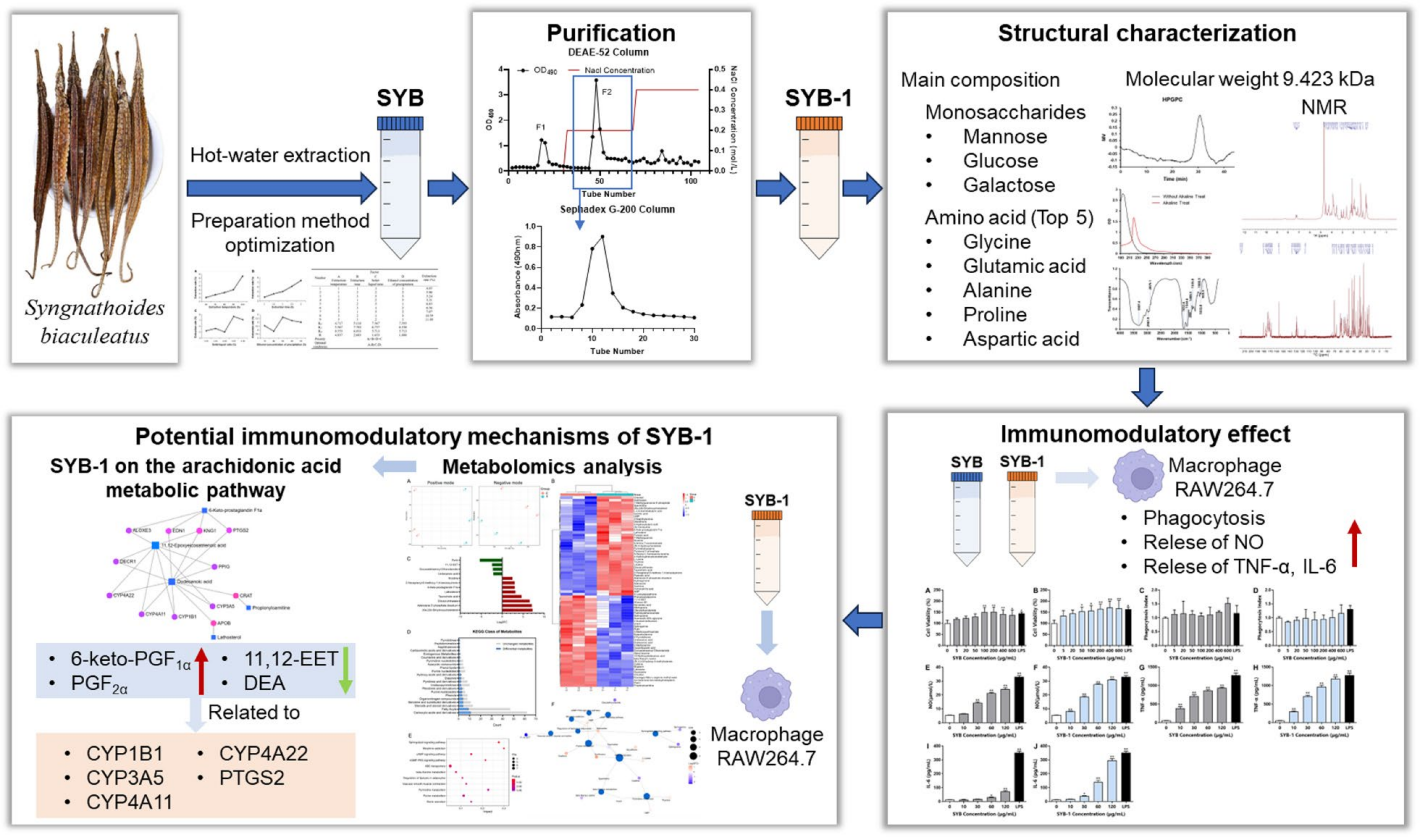

**Supplementary Information:**

The online version contains supplementary material available at 10.1007/s13659-025-00551-6.

## Introduction

*Syngnathoides biaculeatus*, also known as Hailong or Bloch, is a traditional Chinese medicine (TCM) ingredient with a 600-year history. It is widely distributed in the South China Sea, Japan, Philippines, Indian Ocean, East Coast of Africa, and Australia [1, 2]. According to earlier studies, *S. biaculeatus* may be used in TCM to improve human nonspecific immunity, increase and balance vital energy flows within the body, treat impotence, infertility, asthma, high cholesterol, goitre, and kidney disorders, and more [3]. Currently, the majority of reports on *S. biaculeatus* focus on its geographic distribution, biological behavior, and genetic characteristics. Only one report identified its biochemical composition and found that crude protein was the major component, accounting for 58.9% of dry weight [3]. Three studies evaluated the biological activity of its methanol, ethanol, or aqueous extracts. The methanol and aqueous extracts showed significant in vitro antioxidant activity [2, 4], and the ethanolic extract inhibited melanogenesis [5]. However, these studies did not identify the active ingredients from *S. biaculeatus*, especially the main biochemical composition of proteins.

Protein glycosylation is a common observed posttranslational modification in eukaryotes with major effects on protein folding, stability, conformation, distribution, and biological characteristics [6, 7]. Glycoprotein complexes account for about half of all proteins in the organism. According to certain research, glycoprotein complexes have immunomodulatory properties. There is a growing area of study for enhancing the body's immunity [8]. Glycoproteins derived from marine sources have promising futures and will play a significant role in the development of pharmaceuticals for the treatment of a wide range of illnesses [9]. More efficient glycoproteins must be thoroughly studied in order to promote the further exploitation and use of marine-originated glycoproteins for the fields of functional foods and biopharmaceuticals [10, 11]. However, the marine-originated glycoproteins needed for the activities are largely unexplored. In-depth research of these marine animal glycoproteins would provide original insights into the specific structural characteristics required for the observed functions [12].

In order to clarify the unknown water-soluble bioactive components of *S. biaculeatus*, our research performed hot-water extraction to obtain the water-soluble components, glycoproteins. Therefore, in the present study, purification and physicochemical properties of glycoproteins from *S. biaculeatus* were carried out by column chromatography, FT-IR, Amino acid analyzer, HPGPC, GC–MS, and NMR. Furthermore, in vitro immunomodulatory activity and its potential mechanism of the glycoprotein were evaluated on the macrophage RAW264.7. This study is essential in understanding the function of glycoproteins derived from *S. biaculeatus* in the biopharmaceutical and food industries, providing evidence to clarify their bioactive substance.

## Materials and methods

### Materials and reagents

The *S. biaculeatus* was purchased from Zhangshu Qingren Traditional Chinese Medicine Pieces Co., Ltd., Guangzhou, Guangdong, China (Batch No: 201701503). RAW264.7 murine macrophage cells were obtained from Jinan University (Guangzhou, China). Diethylaminoethyl (DEAE)-Cellulose-52 and Sephadex G-200 were obtained from Shanghai Yuanye Bio-Technology Co. Ltd. (Shanghai, China). Glucose, mannose, galactose, inositol, and trifluoroacetic acid (TFA) were purchased from Aladdin Reagent Co., Ltd. (Shanghai, China). Dulbecco Modified Eagle Medium (DMEM) and fetal bovine serum (FBS) were purchased from Gibco Technologies (Grand Island, NY). Lipopolysaccharide (LPS) was purchased from Sigma Co. Ltd. (St. Louis, MO). NO kit and ELISA kits for TNF-*α* and IL-6 were obtained from Neobioscience Co., Ltd. (Shenzhen, China). All other chemical reagents were of analytical reagent grade and were obtained from recognized commercial sources.

### Extraction of glycoproteins and optimization of extraction

#### Single-factor test

Glycoproteins were extracted from *S. biaculeatus* by hot water as described below. Parameters optimized by one-factor test included extraction temperatures (60, 70, 80, 90, 100 ℃), extraction times (1, 1.5, 2, 2.5, 3 h), solid–liquid ratio (1:10, 1:15, 1:20, 1:25, 1:30 g/mL, w/w), and ethanol concentration of precipitation (75, 80, 85, 90, 95%). When the optimization of a particular parameter was carried out, the other parameters were kept constant as extraction temperature 90℃, extraction time 2 h, ethanol precipitation concentration 95%, and solid–liquid ratio 1:20 g/mL (w/w). In brief, dried *S. biaculeatus* (100 g) was crushed into powder. Samples were extracted twice with deionized water for different extraction times at different solid–liquid ratios and temperatures, then concentrated at 60 °C using a vacuum rotary evaporator (Tokyo, Japan). The supernatants were then collected by centrifuging at 12,000 × g for 15 min. Different quantities of cold ethanol (triple volume) were used to precipitate the glycoproteins, which were then left to stand at 4 °C for 12 h. The precipitate was followed by dissolution in water. Finally, crude S. biaculeatus glycoprotein (SYB) was obtained by lyophilizing the solution after it had been dialyzed (8 kDa cut-off membrane) with deionized water for 72 h.

#### Orthogonal test

Based on the results of the Single-factor test, an orthogonal experiment was designed. The extraction rate of SYB was tested by L_9_ (3^4^) orthogonal test using extraction temperature (A, 80, 90, 100 ℃), extraction times (B, 2, 2.5, 3 h), solid–liquid ratio (C, 1:20, 1:25, 1:30 g/mL, w/w), and ethanol concentration of precipitation (D, 85, 90, 95%) as the factors to determine the conditions for the optimal extraction rate.

### Purification of glycoprotein

**Fig. 1:**
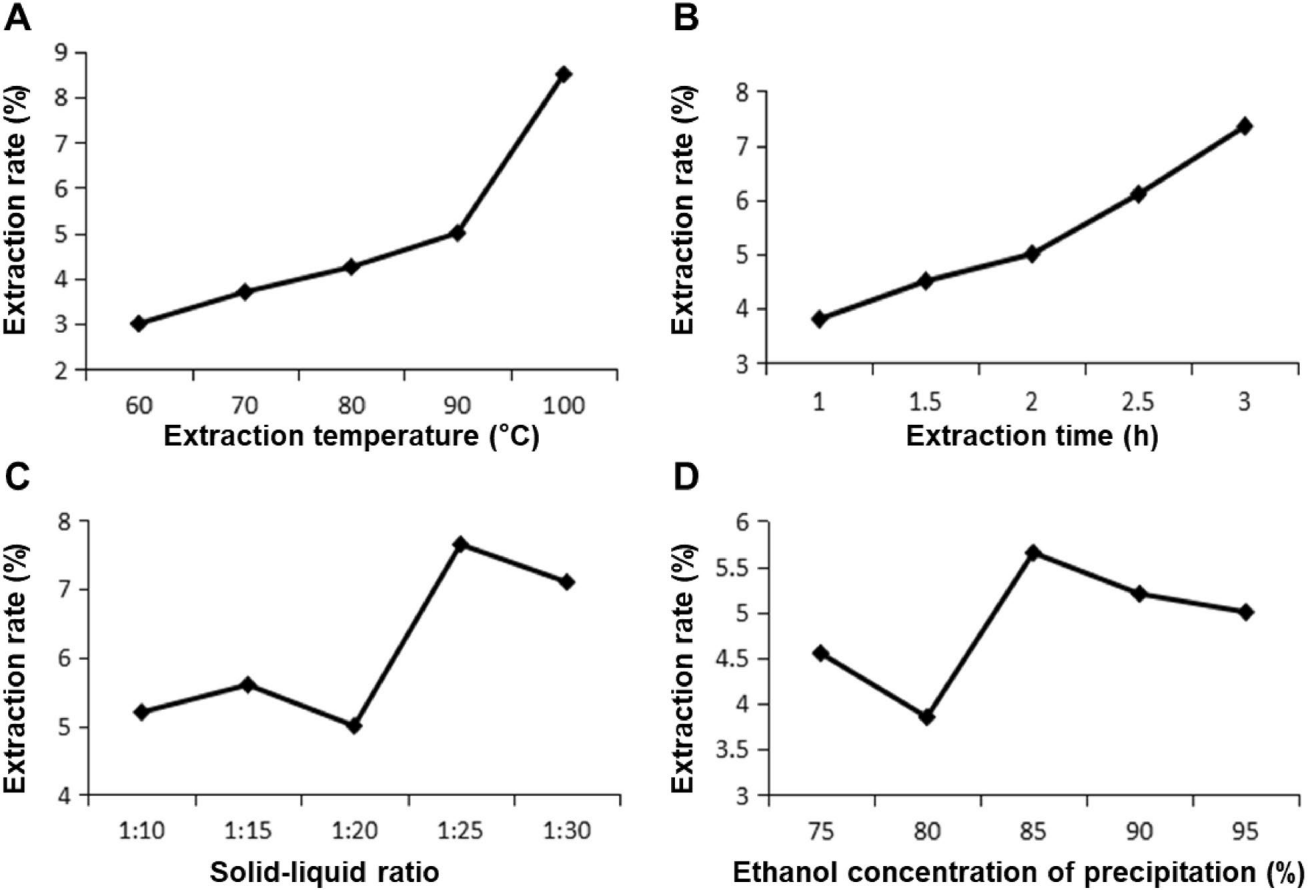
Optimization of extraction rate in a single-factor experiment. **A** Extraction temperature. **B** Extraction time. **C** Solid–liquid ratio. **D** Ethanol concentration of precipitation

Glycoprotein was purified by referring to previously reported methods [13]. SYB water solution at a concentration of 10 mg/mL was loaded onto a DEAE-Cellulose-52 column (3.0 × 50 cm^2^). The flow rate was 1.0 mL/min, and the loading volume was 15 mL. Then it was sequentially eluted in order by distilled water and NaCl solutions (0.2 and 0.4 M). Sevage method removed the free proteins. The phenol–sulfuric acid method was performed to evaluate the carbohydrate content of the eluate fractions [14]. Two peaks were detected under elution with pure water and 0.2 M NaCl solution, respectively (Fig. 1A). The fraction eluted by 0.2 M NaCl solutions (F-2) was concentrated by a rotary vacuum evaporator at 55 °C, then dialyzed (MW cut off 8.0 kDa) at 4 °C for 48 h and lyophilized. Sephadex G-200 column (3.0 × 70 cm^2^) was used to purify further the fraction. The purified fraction was collected and lyophilized, named as SYB-1.

### Physicochemical properties of SYB-1

#### Chemical analysis

Using D-glucose as the reference, the phenol–sulfuric acid method was utilized to determine the total carbohydrate content of SYB-1. Using bovine serum albumin as the reference, the Bradford method was used to assess the protein content [15].

#### Molecular weight determination

Using a Waters HPLC system, the average molecular weight of SYB-1 was determined on HPGPC. The system was equipped with two tandem columns, a TSK-GEL G-5000 PWXL column (10 μm, 7.8 mm × 300 mm inner diameter) and a TSK-GEL G-3000 PWXL column (6 μm, 7.8 mm × 300 mm inner diameter). The detector was Waters 2410 differential refractive index detector. The column temperature was kept at 35 °C while 0.02 mol/L KH_2_PO_4_ was eluted at a flow rate of 0.6 mL/min. SYB-1 solution dissolved in mobile phase was filtered through a 0.45 μm microporous filtration membrane at a concentration of 2mg/mL, and then analyzed by the GPC system. Calibration curves were created using eight distinct molecular weight dextran reference products that ranged from 1 × 10^3^ to 1 × 10^7^ Da [16]. The retention time was used to determine the molecular weight of SYB-1.

#### Infrared spectrum analysis

A Fourier transform infrared (FT-IR) spectrophotometer (Bruker, Ettlingen, Germany) was used to identify the organic functional groups of SYB-1 (2–3 mg) in the 400–4000 cm^−1^ vibrations region [17, 18]. SYB-1 was sufficiently dried and crushed with 100 mg of spectroscopic grade KBr powder (100 mg) before being compressed into a 1 mm pellet for FT-IR analysis using a VERTEX 70 FT-IR infrared spectrometer (Germany Bruker).

#### Monosaccharide composition analysis

The monosaccharide composition of SYB-1 was determined by GC–MS (Agilent, USA) with a TR-5MS capillary column (60 m × 0.25 mm × 0.50 μm). Add 4 mL of 2 M TFA to 10 mg of SYB-1 and hydrolyze at 121 °C for 4 h. After hydrolysis was complete, excess trans fatty acids were removed using nitrogen, and methanol was added in three additions. The hydrolysates were supplemented with pyridine (2 mL) and hydroxylamine hydrochloride (10 mg). Following 40 min of incubation at 90 °C, a total of 2mL acetic anhydride was added and incubated for 30 min. To stop the reaction, 2 mL of water was added. Then extracted three times with 3 mL of chloroform, all chloroform extracts were combined and re-extracted with 6 mL of water. Upon the collection and concentration of the chloroform fraction at 40 °C, the dried samples were dissolved in distilled water that had been ready for system injection.

#### Amino acid composition analysis

The amino acid composition of SYB-1 was detected by an Amino acid analyzer (L-8900, Hitachi, Japan). A total of 50 mg SYB-1 was hydrolyzed with 5 mL HCl (6 M) and vented under nitrogen gas for 2 min, then at 110 °C for 24 h. Hydrolyze fraction was collected and concentrated at 100 °C. Excess HCl was removed by using the addition of ddH_2_O three times [19]. Finally, the hydrolyzate was adjusted to 5 mL with water, filtered through a 0.45 μm microporous membrane, and prepared for injection into the system.

#### Analysis of glycosidic linkage

A sample of 2 mg SYB-1 was dissolved in 4 mL NaOH solution (0.2 mol/L) in a 45 °C water bath for 2 h. The UV scanning was performed at 190–400 nm with NaOH solution (0.2 mol/L) as the reference solution. At the same time, the same concentration of SYB-1 solution without alkaline treatment was measured, distilled water was used as a reference solution, and UV scanning was performed at a range of 190 nm to 400 nm to compare the changes in ultraviolet scanning before and after the *β*-elimination reaction.

#### NMR analysis

NMR analysis was performed referring to a previous report [20]. In order to replace exchangeable protons with D_2_O, SYB-1 was lyophilized three times after being dissolved in D_2_O (99.9% D) at a concentration of 60 mg/mL. NMR analyses were performed at 298 K with a 600 MHz Bruker Advance spectrometer (Fallanden, Switzerland) in the FT mode at 60 °C.

### Assay of immunological activities in vitro

#### Cell viability assay

The macrophage cell line RAW264.7 was maintained in DMEM with 10% (v/v) FBS in a humidified incubator at the conditions of 5% CO_2_ and 37 °C. The effect of SYB and SYB-1 on cell viability was evaluated by CCK-8 assay. In brief, the cells were adjusted to 1 × 10^5^ cells/mL, and 100 μL per well of cell suspension was plated in a 96-well plate and incubated for 24 h. The culture medium was replaced by 100 μL of medium with SYB and SYB-1 (0, 5, 20, 50, 100, 200, 400, and 600 μg/mL). After incubation for 24 h at 37 °C, 10 μL CCK-8 solution was added to each well, and the plate was further incubated for 4 h at 37 °C [21]. Subsequently, a microplate reader was used to measure the absorbance at 450 nm.

#### Phagocytosis assay

The effects of SYB and SYB-1 on phagocytic activity were evaluated by neutral red phagocytosis assay. In brief, the SYB and SYB-1 treatment groups refer to the procedure in 2.5.1. The difference is an additional positive control group, treated with LPS (1 μg/mL). After 24 h of treatment, the suspension was removed, and 100 μL neutral red solution (1 μg/mL) was added to each well, followed by 1 h of incubation. After decanting the neutral red solution, the cells were washed with PBS twice. Finally, 100 μL of cell lysis buffer (acetic acid/ethanol = 1:1, v/v) was added to each well. The absorbance of the mixture at 540 nm was measured after the mixture had been incubated at room temperature for 12 h [22]. The results were corrected with cell viability values.

#### Measurement of cytokine and NO production

In brief, 2.5 × 10^5^ cells per well of RAW264.7 cells were plated in a 24-well plate and incubated for 24 h. The culture medium was replaced by 1000 μL of SYB or SYB-1 solutions (0, 10, 30, 60, and 120 μg/mL) in a new medium, respectively. LPS in a new medium (1 μg/mL) was used as a positive control. After incubation for 24 h at 37 °C, the suspension was collected for measurement by NO concentration kits and ELISA kits of TNF-*α* and IL-6 according to the manufacturer's protocol.

### Metabolomics study of SYB-1 in RAW264.7

The Metabolomics of SYB-1 on RAW264.7 cells was treated by two groups of control (C group) and SYB-1-treated groups (T group), with 3 replicates per group. C group was treated with blank medium, and T group with 120 μg/mL SYB-1.

A HILIC column in an ultra-high performance liquid chromatography system (UHPLC; Agilent, USA) was used to separate the samples. Samples were put in an autosampler (4 °C). Furthermore, to track and assess the system's stability and the experiment's dependability, the quality control was added at random into the sample queue. Following separation, the samples were examined using an AB Triple TOF 6600 mass spectrometer (SCIEX, USA) and identified using electrospray ionization (ESI) in both positive and negative ion modes.

Upon peak alignment, XCMS software was used to recover the peak area of the original data and correct the retention time. After that, metabolite structural identification, data pre-processing, and quality assessment were carried out. The PCA and OPLS-DA were performed with the ropls package in R to calculate the variable importance in the projection (VIP) value. The metabolites were classified as significant differential accumulated metabolites (DAMs) while the VIP value was greater than 1 and the *p*-value was less than 0.05. Subsequently, KEGG pathway analysis and network analysis were carried out using MetaboAnalyst v5.0.

### Statistical analysis

Data were expressed as means ± standard deviations, and one-way analysis of variance ANOVA followed by Tukey’s test. Statistical analyses were performed using SPSS software version 20.0 or GraphPad Prism 8.

## Results and discussion

### Optimization of the extraction of SYB

#### Optimization of extraction rate in single-factor experiment

It takes a certain amount of time to reach equilibrium in the extraction process when extracting active ingredients from products. Therefore, in this study, the parameters of its extraction method were first optimized. The temperature during extraction had a major impact on the rate at which SYB was extracted (Fig. 1A). As shown in Fig. 1B, it illustrated that the extraction rate of SYB rose progressively with the increase in time and reached the maximum at 2–3 h. For the parameters of extraction temperature, the extraction rate of SYB showed a tendency to increase with temperature, and was best at 100 °C. The solid–liquid ratio is an important factor in the effectiveness of SYB extraction since it influences the area of contact between the solvent and the raw material. It is clear from Fig. 1C that the extraction rate of SYB increased about 1.5-fold at 1:25 g/mL compared to 1:20 g/mL. On the other hand, the extraction rate slightly decreased with a gradual increase in concentration from 1:25 to 1:30 g/mL. The alcohol precipitation concentration mentioned is the concentration of ethanol added to the mixed solution of the SYB during the concentrated alcohol precipitation operation. Under alcohol precipitation concentration conditions, with the increase in alcohol precipitation concentration, the extraction rate of SYB presented a trend of first decreasing, then increasing, and finally decreasing, with a peak at 85% (Fig. 1D).

Therefore, the conditions for the optimal extraction rate of SYB by single-factor test were extraction temperature of 100 ℃, time of 3 h, alcohol precipitation concentration of 85%, and solid–liquid ratio of 1:25 (g/mL).

#### Optimization analysis of orthogonal experiment

The parameters of extraction temperature (A, 80, 90, 100 ℃), extraction time (B, 2, 2.5, 3 h), solid–liquid ratio (C, 1:20, 1:25, 1:30 g/mL, w/w), and ethanol concentration of precipitation (D, 85, 90, 95%) were further optimized by orthogonal experimental design L_9_ (3^4^). As the results showed in Table 1, factors influencing the extraction rate of SYB were ranked in the order of temperature, extraction time, alcoholic precipitation concentration, and solid–liquid ratio. The optimal condition was the extraction temperature of 100°C, extraction time of 2.5 h, solid–liquid ratio of 1:20 g/mL, and alcohol precipitation concentration of 85%, which resulted in an extraction rate of 11.0% for the SYB.

**Table 1:** Orthogonal experimental design L_9_ (3^4^) to assess the effects of extraction temperature, extraction time, solid–liquid ratio, and ethanol concentration of precipitation on the extraction rate

Number	Factor				Extraction rate (%)
	A Extraction temperature	B Extraction time	C Solid–liquid ratio	D Ethanol concentration of precipitation	
1	1	1	1	1	4.95
2	1	2	2	2	5.96
3	1	3	3	3	3.24
4	2	1	2	3	3.31
5	2	2	3	1	6.83
6	2	3	1	2	6.56
7	3	1	3	2	7.07
8	3	2	1	3	10.59
9	3	3	2	1	11.00
K_1_	4.717	5.110	7.367	7.593	
K_2_	5.567	7.793	6.757	6.530	
K_3_	9.553	6.933	5.713	5.713	
R	4.837	2.683	1.653	1.880	
Priority	A > B > D > C				
Optimal conditions	A_3_B_2_C_1_D_1_				

### Physicochemical properties of SYB-1

#### Purification of the SYB

**Fig. 2:**
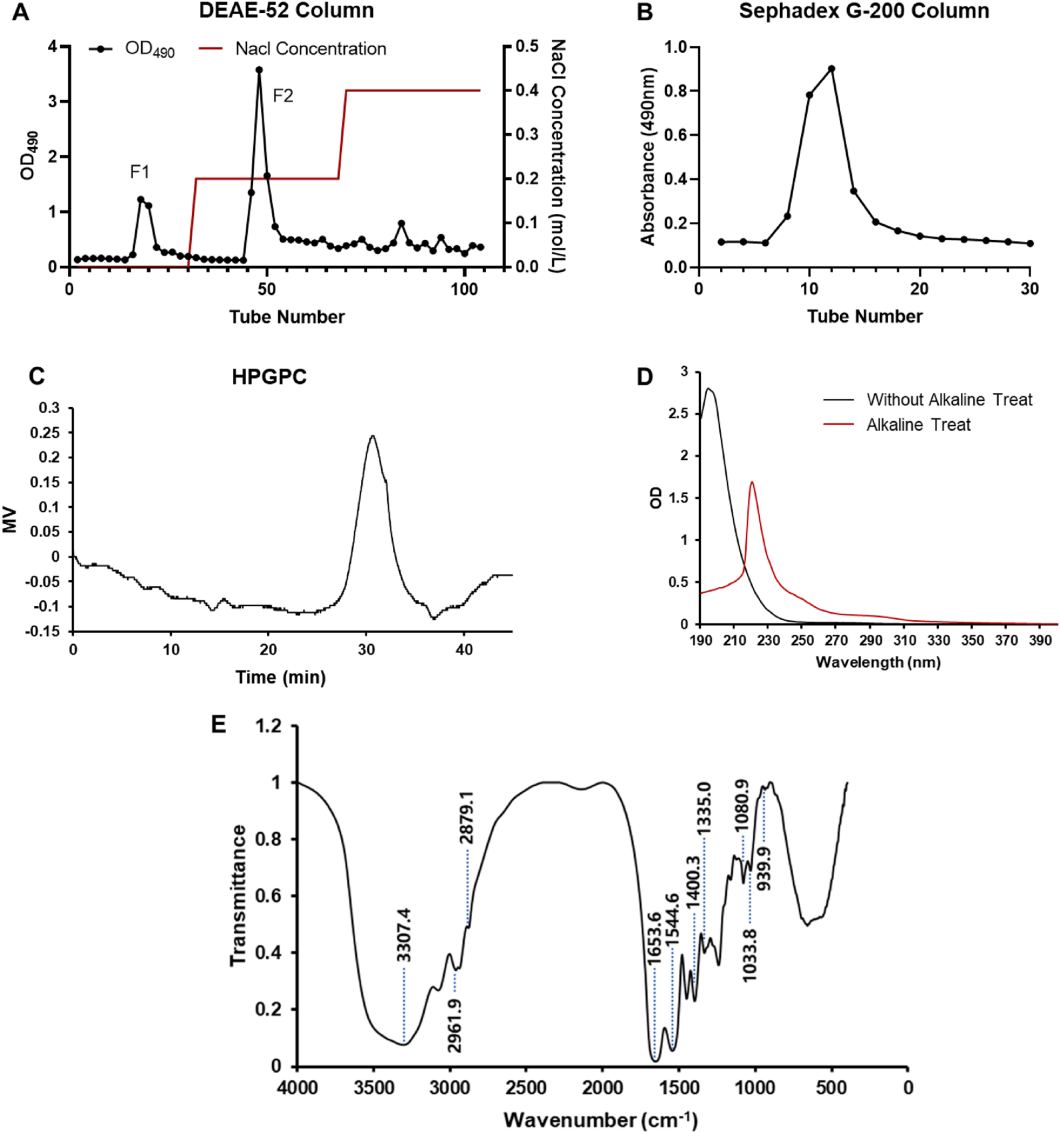
The preparation and physicochemical properties of SYB-1. **A** Crude SYB elution profile on a DEAE-52 column. **B** Purification of SYB-1 on a Sephadex G-200 column. **C** HPGPC profile. **D** The UV spectrum. **E** The IR spectrum

Crude SYB was separated on a DEAE-52 column and showed high elution peaks in 0 and 0.2 M NaCl buffers (Fig. 2A). The primary glycoprotein fraction was eluted by 0.2 M NaCl (F2). In the present study, the fraction F2 was further purified by Sephadex G-200 column. It was a single elution peak as shown in Fig. 2B. In subsequent studies, we focused on this single peak fraction, which is named SYB-1.

#### Composition, homogeneity, and molecular weight of SYB-1

The saccharide content in SYB-1 was 8.46% by the phenol–sulfuric acid method, and protein content was 33.37% by the Bradford method. It was suggested that SYB-1 belonged to a kind of glycoprotein that employed protein as the backbone and branched with saccharide chains. HPGPC analysis showed that there was only one main single peak, with a molecular weight of 9.423 kDa (Fig. 2C).

The chemical composition and chain component of the glycoconjugate are important for their application and bioactivities1. Three different types of monosaccharides—mannose, glucose, and galactose—with molar ratios of 1.00: 1.34: 2.09 were found to make up SYB-1, according to GC–MS analysis, with galactose being the most common kind (Figure S1).

Amino acid analysis provides amino acid composition and is considered to be the gold standard for quantitative protein content directly [23]. Table 2 showed that the amino acid composition of SYB-1 was seventeen amino acids determined by amino acid analysis. The major amino acids were glycine (19.68 g/100 g), followed by glutamic acid (10.64 g/100 g), alanine (9.36 g/100 g), proline (8.90 g/100 g), and aspartic acid (8.51 g/100 g). Since glutamic acid and aspartic acid rank high, SYB-1 should be a polysaccharide-binding acidic protein. The total amino acid content is 88.81 g/100g (88.81%), which is equivalent to the total protein content [24]. However, the total amino acid content differs from the protein content of 33.37% measured by the Bradford method. This discrepancy is mainly due to the limitations of semi-quantitative methods, which are affected by differences in the amino acid composition of the standard and sample, potentially leading to inaccurate estimates of protein content. In the two current results, we use the total amino acid content of 88.81% determined by amino acid content measurement as a more accurate reference for more protein content. Based on the result of total amino acid and saccharide contents, the glycoprotein component of SYB-1 was estimated to be 97.27%.

**Table 2:** Amino acid composition of SYB-1

Name	SYB-1 (g/100 g)
Glycine	19.68
Glutamic acid	10.64
Alanine	9.36
Proline	8.90
Aspartic acid	8.51
Arginine	7.45
Lysine	4.03
Serine	3.26
Threonine	3.14
Leucine	3.05
Valine	3.02
Phenylalanine	2.39
Isoleucine	1.74
Methionine	1.58
Histidine	1.08
Tyrosine	0.90
Cysteine	0.08
Total amino acid	88.81

#### Linkage analysis of SYB-1

As shown in Fig. 2D, UV spectra before and after the alkaline treatment of SYB-1 were obtained. Both showed no absorption at 260 nm, but rather a weak peak at 280 nm after alkali treatment compared to before. It could be concluded that there was no nucleic acid, only some protein. Following alkaline treatment, SYB-1 showed a greater absorbance at 240 nm, indicating the presence of an O-glycopeptide bond. Glycoproteins can be linked in a number of ways using distinct amino acids that are frequently present in many known glycoproteins. For example, N-glycosylation and O-glycosylation only happen through GalNAc-*α*-Serine/Threonine links, respectively. N-glycopeptide linkage and O-glycopeptide linkage are the two primary forms of glycopeptide linkage found in polysaccharide-protein (peptides), based on their alkaline stability. Since the serine and threonine of glucosidic bonds translated into *α*-aminoacrylic acid and *α*-aminocrotonic acid, respectively, the N-glycopeptide linkage always stays stable under low-concentration alkaline conditions, while the other is easily broken and subsequently yields obvious absorption at 240 nm, according to the *β*-elimination reaction.

Galactose was the most abundant monosaccharide in the chemical compositions of SYB-1, while amino acid analysis revealed comparatively high levels of glutamic acid and glycine (Table 2). It was demonstrated that O-glycosidic linkages, which may include O-glycosylation, were implicated in the binding of protein to carbohydrate [25]. It includes O-Glc, O-Man, O-Xyl, O-Gal, O-Fuc, O-GlcNAc, and O-GalNAc. In this study, we found that SYB-1 contains monosaccharides mannose, glucose, and galactose, and amino acids threonine and serine. Thus, there may be O-GalNAc and O-GlcNAc glycosylation sites in this glycoprotein.

#### FT-IR spectra of SYB-1

The SYB-1 FT-IR spectra revealed several absorption bands that appeared following wave numbers and were attributed according to previous reports (Fig. 2E) [9, 26]. Hydrogen-bonded hydroxyl groups were identified as the source of a wide band at 3307.8 cm^−1^ [27]. C-H stretching caused a strong band at about 2962.5 cm^−1^ and 2879.6 cm^−1^. The presence of protein was further demonstrated by the comparatively strong absorption bands at 1653.6 cm^−1^ and 1544.6 cm^−1^, which were ascribed to the carbonyl bond of the amide group [9]. Pyran glycosides should be identified by their unique IR absorption bands at 1080.9 cm^−1^ and 1033.8 cm^−1^, while mannose and methylene of deoxysugar should be identified by their absorption bands at 939.9 cm^−1^. The presence of carboxyl (-COO-) groups was indicated by the high absorption peak at around 1400.3 cm^−1^ and the weak one at about 1335 cm^−1^ [28]. Consequently, there were two types of end carbon-glucoside bonds (*α*- and *β*-configurations). The typical absorption at 820 cm^−1^ indicated that the bond in SYB-1 was *α*-style.

#### NMR analysis

**Fig. 3:**
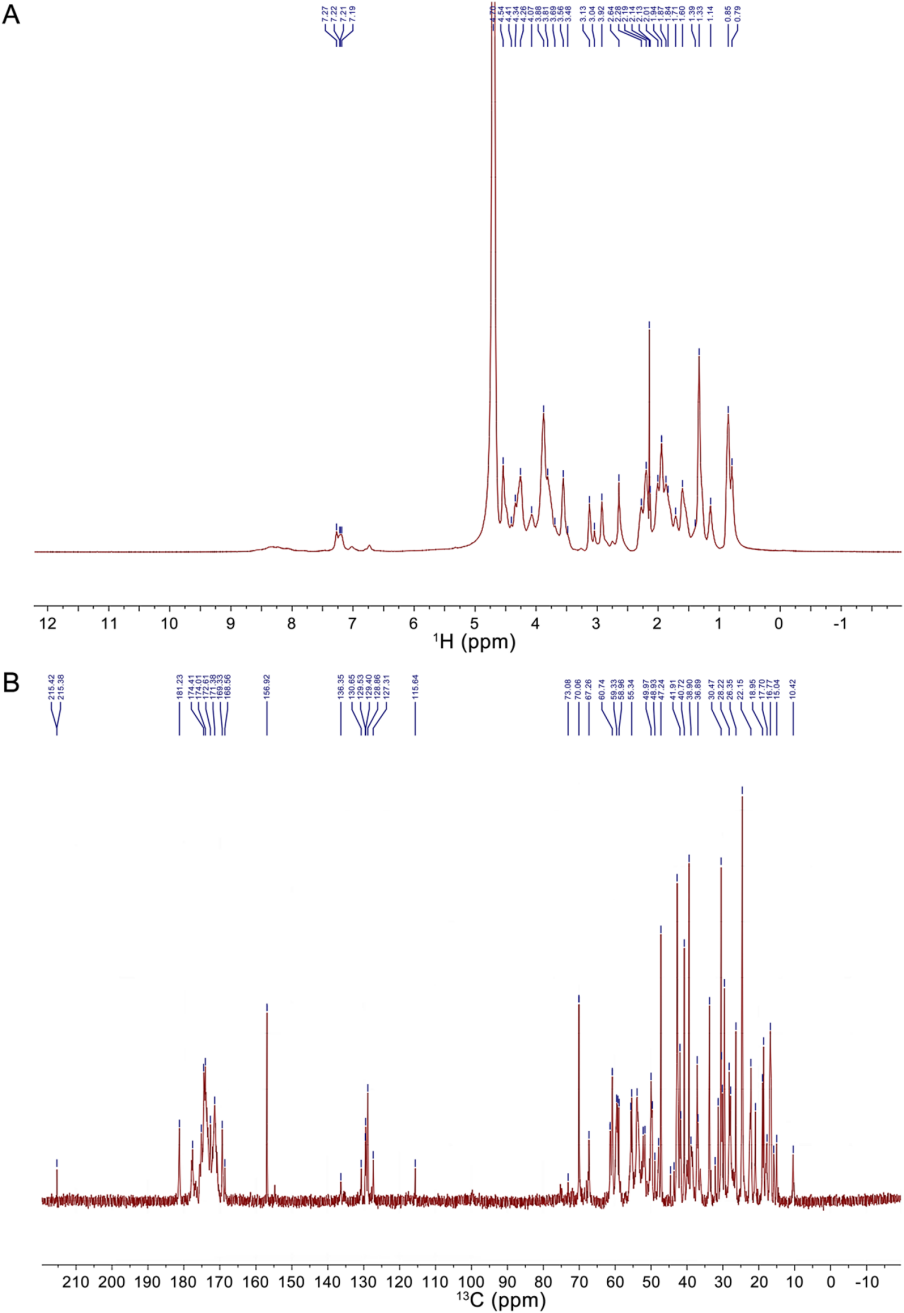
NMR spectra of SYB-1. **A** ^1^H NMR. **B** ^13^C NMR

The ^1^H and ^13^C NMR spectra provide critical insights into the structural properties of SYB-1. The signal assignment of amino acid residues in the ^1^H and ^13^C NMR spectra (Fig. 3) took advantage of Biological Magnetic Resonance Data Bank (BMRB) [29], and the results of signal attribution are shown in Tables 3, 4.

**Table 3:** Proposed assignment of ^1^H NMR signal

Observed *δ* (ppm)	Residue	Atom	BMRB reference (ppm)
7.27–7.19	Phenylalanine/Tyrosine	H*δ*/Hε	7.04–7.43
4.54	Aspartic acid	H*α*	4.582
4.41	Serine	H*α*	4.474
4.34	Proline	H*α*	4.385
4.26	Lysine	H*α*	4.256
0.85/0.79	Valine	Met-ε-CH₃	0.818/0.802

**Table 4:** Proposed assignment of ^13^C NMR signal

Observed *δ* (ppm)	Residue	Atom	BMRB reference (ppm)
177.6	Alanine	C = O	177.745
174.6	Threonine	C = O	174.459
174.41	Serine	C = O	174.553
172.61	Glycine	C = O	173.830
70.06	Threonine	C*β*	69.604
61.42	Threonine	C*α*	62.195
58.96	Serine	C*α*	58.676
55.63	Aspartic acid	C*α*	54.669
53.84	Alanine	C*α*	53.144
33.71–30.38	Arginine	C*β*	30.668
22.15–18.62	Alanine	C*β*	19.026

In the ^1^H spectrum, the signal at *δ* 4.70 ppm is the solvent peak of deuterated water D_2_O. The signals from *δ* 9.0–7.1 ppm were derived from the backbone signals of protein, while *δ* 7.5–6.0 ppm originated from the side chains of protein [30, 31]. The chemical shift at *δ* 7.19–7.27 ppm can be attributed to the benzene ring of phenylalanine or tyrosine residues on the protein backbone or side chain [32]. The chemical shift at *δ* 4.54, 4.41, 4.34, 4.26 is attributed to H*α* of aspartic acid, serine, proline, and lysine, respectively. The aliphatic region (*δ* 0–3 ppm) shows diagnostic methyl groups of valine (*δ* 0.85–0.79 ppm) and arginine *β*-methylene (*δ* 2.92 ppm) [30]. The structure of the glycan residues is *β*-linked due to the chemical shift at *δ* 4.8–4.4 ppm, while there is no chemical shift at *δ* 5.3–4.9 ppm for *α*-linked residues [28]. However, these signals may overlap with the H*α* signals of amino acids. The peaks in the *δ* 3.0–4.2 region were assigned to the protons of carbons C-2 to C-5 (or C-6) of the glycosidic rings [33].

The ^13^C spectrum (Fig. 3B) reveals carbonyl signals at *δ* 177.60 (alanine C = O), *δ* 174.60 (threonine C = O), *δ* 174.41 (serine C = O), and *δ* 172.61 ppm (glycine C = O) [32], with aromatic carbons at *δ* 130–127 ppm confirming phenylalanine presence [27]. Protein secondary structure elements are indicated by *α*-helix-associated carbons (threonine C*α* at *δ* 61.42, serine C*α* at *δ* 58.96 ppm, aspartic acid C*α* at *δ* 55.63 ppm) and *β*-sheet domains (arginine C*β* at *δ* 33.71–30.38 ppm, alanine C*β* at *δ* 22.15–18.62 ppm). Other signals at *δ* 80–60 ppm can be attributed to the carbon signal on the glycan residue C2-C6 [34]. Notably, our amino acid composition includes threonine and serine, which are two possible sites for O-glycosylation, resulting in a downfield shift (> 3 ppm) for threonine C*β* and an upfield shift (< -3 ppm) for serine C*β* [35]. In the BMRB data, the signals for non-glycosylated threonine and serine C*β* appear at *δ* 69.604 and *δ* 63.723 ppm, respectively, and our results detected a signal (*δ* 70.06 ppm) that may be attributed to threonine C*β*. Additionally, signals indicating an upfield shift for threonine C*β* (*δ* 73.08 ppm) and a downfield shift for serine C*β* (*δ* 60.74 ppm) were detected, suggesting the presence of O-glycosylation.

The NMR results align with the amino acid composition of SYB-1, particularly high-abundance residues (Gly, aspartic acid, alanine, arginine), confirming its glycoprotein properties with predominant *β*-linked glycans and an O-glycosylation site.

### Immunomodulatory activity in vitro of SYB and SYB-1

#### Effects of SYB and SYB-1 on cell viability and phagocytosis

**Fig. 4:**
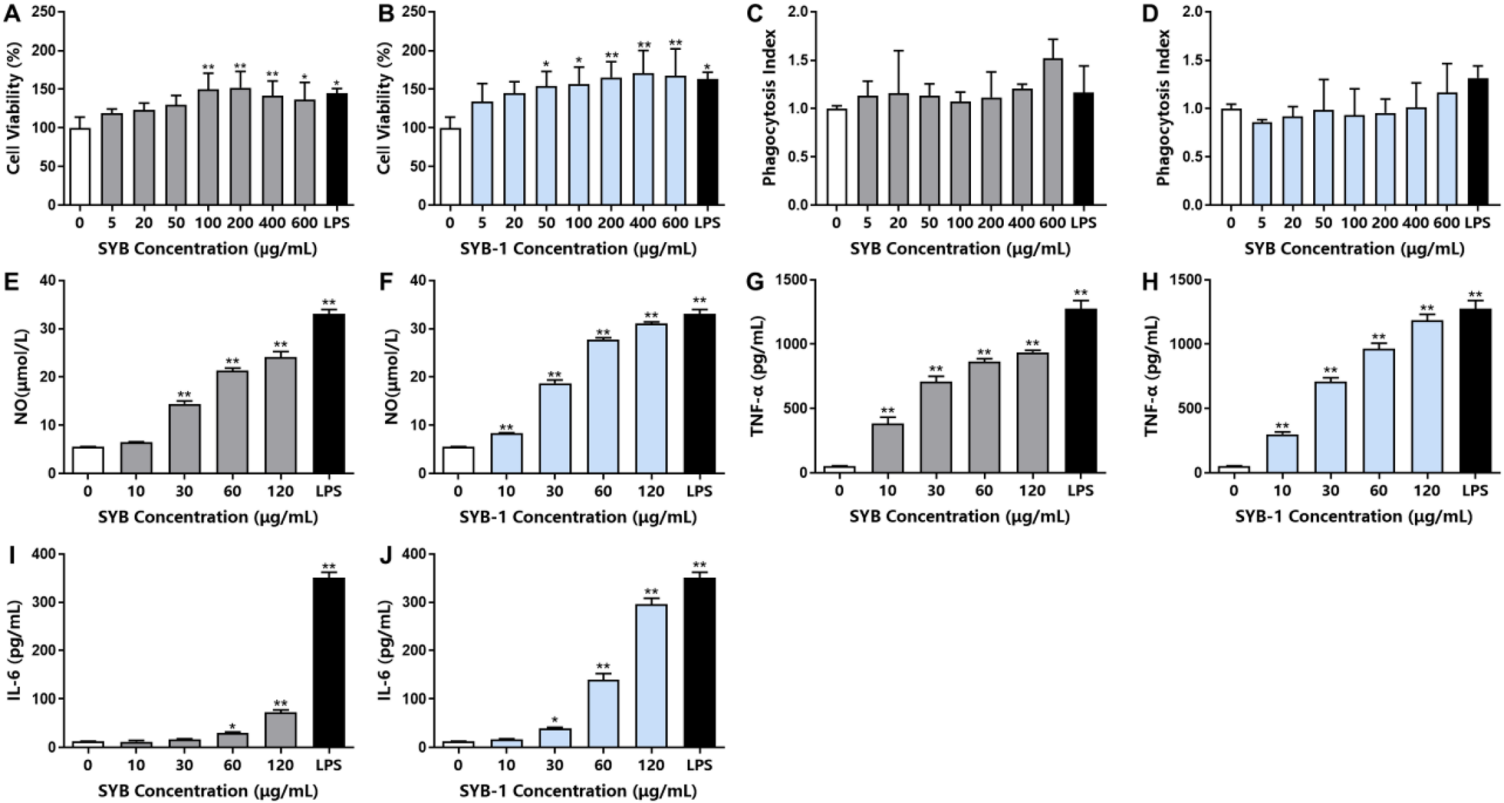
Immunomodulatory activity of SYB and SYB-1 on macrophages RAW 264.7 in vitro. **A**, **B** Cell viability: SYB (**A**), SYB-1 (**B**). **C**, **D** Phagocytosis index: SYB (**C**), SYB-1 (**D**). **E**, **F** NO release: SYB (**E**), SYB-1 (**F**). **G**, **H** TNF-*α* release: SYB (**G**), SYB-1 (**H**). **I**, **J** IL-6 release: SYB (**G**), SYB-1 (**H**). * and ** represent *p* < 0.05 and *p* < 0.01 compared with control, respectively

The immunomodulatory effect is one of the more common biological activities of glycoproteins [36]. To investigate the immunomodulatory effect of SYB and SYB-1 on the RAW264.7 cells, the cell viability was evaluated by CCK8 assay, and phagocytosis was detected by neutral red phagocytosis assay with correction by cell viability. The cell viability ratio of different treated concentrations showed a rising tendency, indicating that SYB and SYB-1 under 600 µg/mL were nontoxic to RAW264.7 cells (Fig. 4A, B). At 200 µg/mL, SYB reached its peak of proliferation, whereas SYB-1 did the same at 400 µg/mL. So, the concentration of SYB and SYB-1 used in the following study was below 600 µg/mL. As illustrating in Fig. 4C, SYB demonstrated boosting effects on macrophage phagocytosis without significant changes in the dose range of 5–600 μg/mL. For SYB-1, in the dose range of 0–400 μg/mL, the effect on phagocytosis was decreased or unchanged (Fig. 4D). Only at 600 μg/mL phagocytosis was increased. However, none of these changes were significant.

#### Effects of SYB and SYB-1 on the production of NO and cytokines

Macrophages play a key role in the innate and adaptive immune responses by producing chemicals that have immunomodulatory, tumoricidal, and microbicidal effects [37]. When macrophages are activated in the presence of an exogenous stimulus, they naturally release a substantial amount of pro-inflammatory chemicals, including NO and cytokines (TNF-*α* and IL-6). NO is a highly reactive free radical that is essential to host defense. Therefore, the impact of SYB and SYB-1 on NO, TNF-*α*, and IL-6 levels in RAW264.7 cells was detected.

SYB and SYB-1 were able to induce statistically significant increases (*p* < 0.01) in NO production at most concentrations tested compared to the control (0 μg/mL SYB or SYB-1) (Fig. 4E, F). The production of NO significantly increased at all concentrations of SYB and SYB-1, except for 10 μg/mL SYB. As shown in Fig. 4G–J, the lowest production of TNF-*α* and IL-6 was observed in the blank control. Compared with the control, treatments with various concentrations of SYB or SYB-1 for 24 h significantly stimulated the production of TNF-*α* (10, 30, 60, or 120 μg/mL) and IL-6 (30, 60, or 120 μg/mL) in a dose-dependent manner (*p* < 0.05). The production of cytokine IL-6 was highly increased at the high concentrations of SYB and SYB-1, especially SYB-1 at 120 μg/mL. Therefore, by stimulating the release of cytokines, both SYB and SYB-1 demonstrated significant immunomodulatory effects in RAW264.7.

### Potential immunomodulatory mechanisms of SYB-1 in macrophage RAW264.7

#### Differential metabolite screening and involved pathway analysis

**Fig. 5:**
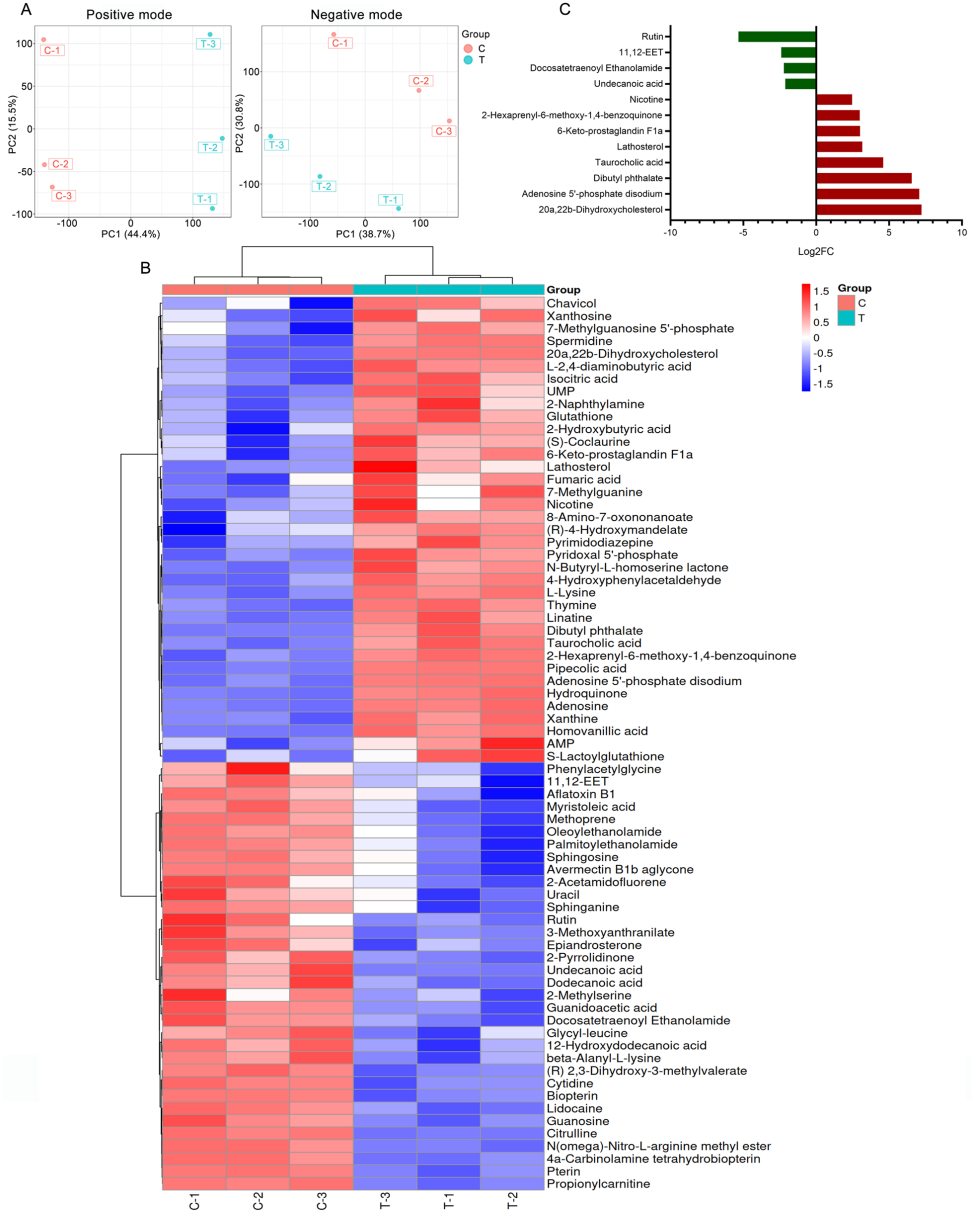
Differentially accumulated metabolites analysis of SYB-1 on RAW264.7. **A** PCA analysis of positive and negative modes. **B** Heatmap of DAMs. **C** The barplot of DAMs with |Log_2_FC| greater than 2

Differentially accumulated metabolites (DAMs) between the SYB-1-treated groups (T group) and the control groups (C group) were investigated. The PCA analysis indicates that SYB-1 treatment greatly altered the metabolites of RAW264.7 cells, which remained stable in the same groups (Fig. 5A). DAMs with significant differences were defined as metabolites with a p < 0.05 and a variable importance for the projection (VIP) value > 1. Metabolites with positive and negative Log_2_FC were found to be up-regulated DAMs and down-regulated DAMs, respectively, following screening under the aforementioned criteria. In all, 71 DAMs were found in this investigation (Fig. 5B), of which 34 were down-regulated, and 37 were up-regulated. Further screening for DAMs with large |Log_2_FC| at a threshold of 2 resulted in eight up-regulated DAMs: 20a,22b-dihydroxycholesterol, adenosine 5'-phosphate disodium, dibutyl phthalate, taurocholic acid, lathosterol, 6-keto-prostaglandin F1a, 2-hexaprenyl-6-methoxy-1,4-benzoquinone, and nicotine; four down-regulated DAMs: Rutin, 11,12-EET, docosatetraenoyl ethanolamide, and undecanoic acid (Fig. 5C).

**Fig. 6:**
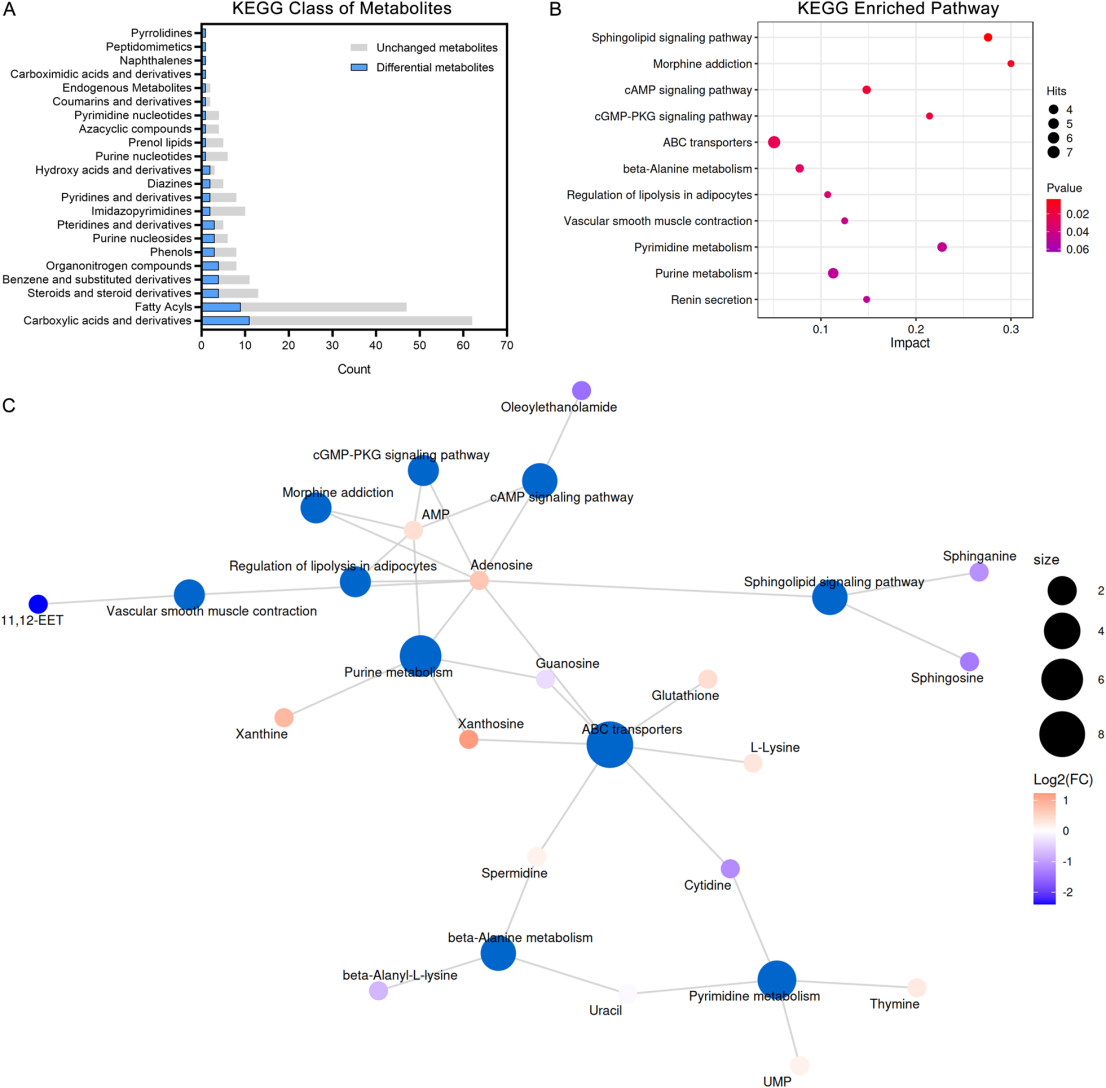
KEGG pathway analysis of SYB-1 on RAW264.7. **A** A statistical plot of the KEGG category's metabolite count. **B** Significantly enriched KEGG pathways with *p* < 0.05. **C** Network of metabolite-pathway relationships

The primary classifications were annotated by the KEGG database. The top five categories with the highest number of DAMs were “Carboxylic acids and derivatives” (11 DAMs), “Fatty Acyls” (9 DAMs), “Steroid and steroid derivatives” (4 DAMs), “Benzene and substituted derivatives” (4 DAMs), and “Organonitrogen compounds” (4 DAMs) (Fig. 6A). The two categories in the above were related to lipids, which were potential key metabolite categories. KEGG enrichment analysis provides access to cellular metabolic pathways affected by SYB-1. The top 20 pathways by p-value were shown in Fig. 6B, and the number of 11 KEGG pathways were significantly enriched (p < 0.05). Adenosine is involved in the most enrichment pathways (9 pathways), followed by AMP with 6 pathways involved (Fig. 6C).

Among the significantly enriched pathways, “cAMP signaling pathway” and “cGMP-PKG signaling pathway” were both closely associated with macrophage immune regulation. These pathways shared two common DAMs, AMP and its downstream metabolite adenosine, which were modestly up-regulated (Log_2_FC = 0.40 and 0.70, respectively). In the “cGMP-PKG signalling pathway”, adenosine is presented as mediating NO synthesis [38]. Correspondingly, our results demonstrated elevated NO levels (Fig. 4F), suggesting a possible link between adenosine accumulation and NO production. Nonetheless, adenosine is generally regarded as immunosuppressive on the activation of most immune cells [39], such as macrophages in response to bacterial infection [40]. This duality complicates the interpretation of its role in the current study. In addition, the “Purine metabolism” pathway, which is one of the upstream pathways for adenosine, included four up-regulated and one down-regulated DAMs. Although purine metabolism is typically associated with immunosuppressive functions, Yang et al. [41] reported that blockade of this pathway could alleviate macrophage immunosuppression. Thus, the functional consequences of enhanced adenosine levels in our context remain inconclusive and warrant further mechanistic investigation.

The other DAM sphinganine from the most significantly enriched KEGG pathway “Sphingolipid signaling pathway”, intriguingly, whose accumulation promotes TLR4 signaling and pro-inflammatory cytokine release [42], was down-regulated in this study (VIP = 1.3226, *p* = 0.0203, Log_2_FC = −1.14). The remaining significantly enriched KEGG pathways were mainly related to DAMs, AMP and adenosine, which could not be further analysed, including “Morphine addiction”, “Regulation of lipolysis in adipocytes”, “Vascular smooth muscle contraction”, and “Renin secretion”. Thus, besides the significantly enriched KEGG pathways, we need to look for other pathways that are consistent with our phenotypic results.

#### Potential effects of SYB-1 on the arachidonic acid metabolic pathway

**Fig. 7:**
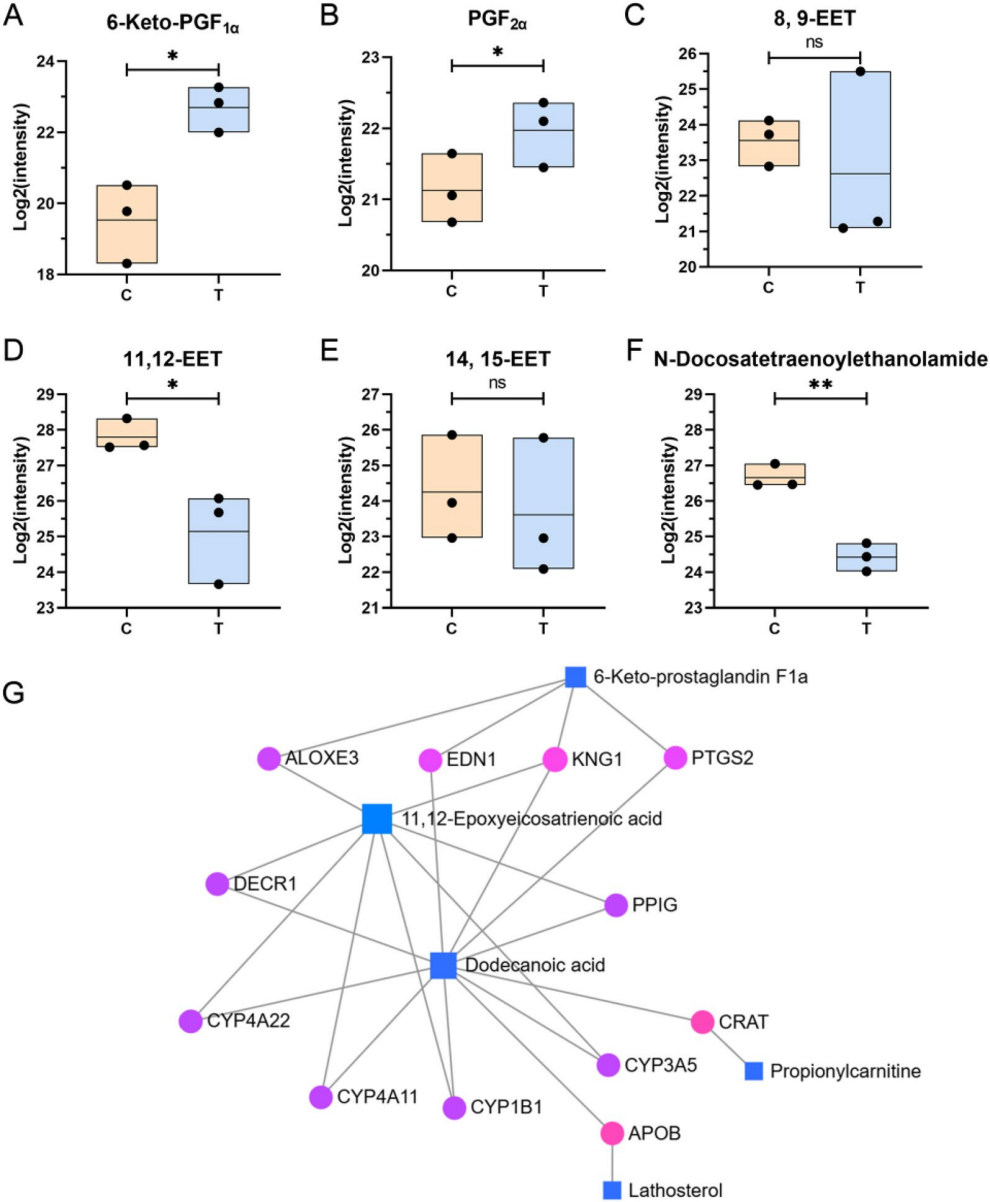
Metabolites associated with arachidonic acid metabolism in metabolomics. **A**–**F** Variations in metabolites associated with arachidonic acid metabolism, including 6-keto-PGF_1*α*_ (**A**), PGF2_*α*_ (**B**), 8,9-EET (**C**), 11,12-EET (**D**), 14,15-EET (**E**), N-Docosatetraenoylethanolamine (**F**). **G** DAMs-gene interaction network. *, **, and ns represent *p* < 0.05, *p* < 0.01 and *p* > 0.05 compared with control, respectively

Since most pathways highlighted by KEGG enrichment could not directly explain macrophage immunomodulation in our results, we instead focused on DAMs with larger fold‑changes (|Log_2_FC|> 2) (Fig. 5C). It is worth mentioning that two DAMs with |Log_2_FC|> 2, involving the arachidonic acid (AA) metabolism pathway, including 6-keto-prostaglandin F1a (6-keto-PGF_1*α*_) (VIP = 1.5705, *p* = 0.0133, Log_2_FC = 3.00) and 11,12-EET (VIP = 1.5186, *p* = 0.0282, Log_2_FC = −2.40). Moreover, 1 DAM N-Docosatetraenoylethanolamide (VIP = 1.4504, *p* = 0.0018, Log_2_FC = −2.23) may be associated with the AA metabolism pathway. Considering the above potential association, we searched for and collated more information on metabolites related to AA metabolism in the metabolomics results (Fig. 7A–F), although some of the metabolites did not show significant changes.

The AA metabolism, initiated by the release of AA, plays a pivotal role in maintaining immune homeostasis [43]. Under physiological conditions, AA is esterified in membrane phospholipids. Upon cellular stimulation—particularly during inflammatory responses—phospholipids are hydrolyzed by phospholipase A_2_ (PLA_2_) and phospholipase C (PLC), resulting in the liberation of free AA from the cell membrane [44]. Following that, AA is metabolized via four major enzymatic pathways: the cyclooxygenase (COX) pathway, the lipoxygenase (LOX) pathway, the cytochrome P450 (CYP450) pathway, and the anandamide pathway [43]. In this study, DAMs associated with the AA metabolic pathway involved 3 synthetic directions: COX, CYP450, and anandamide pathways, suggesting a multifaceted regulatory role of AA metabolism in the observed biological processes.

1. The cyclooxygenase pathway.

The COX pathway plays a central role in the biosynthesis of various prostaglandins (PGs), which are critical lipid mediators regulating a wide range of physiological functions and inflammatory responses [45]. These PGs are synthesized from AA via the enzymatic actions of COX-1 and COX-2, producing the unstable intermediate PGH_2_. PGH_2_ is then converted by specific terminal synthases into major prostanoids, including prostacyclin (PGI_2_), PGE_2_, PGD_2_, PGF_2*α*_, and thromboxane A_2_ (TXA_2_) [46]. Among these, COX-2 is a crucial modulator of immune responses, with its expression and downstream PG products typically upregulated in macrophages upon pro-inflammatory stimulation [47, 48]. As shown in Fig. 7A, B, treatment with SYB-1 upregulated the levels of 6-keto-PGF_1*α*_ and PGF_2*α*_ in macrophages, both of which are derived from PGH_2_. PGI_2_ is enzymatically generated from PGH_2_ by prostacyclin synthase (PGIS), but due to its chemical instability, it rapidly hydrolyzes into the more stable metabolite 6-keto-PGF_1*α*_ [49], which is commonly used as a surrogate marker for PGI_2_ production. PGIS and PGI_2_ have been shown to exert anti-inflammatory effects in macrophages [50]. Notably, PGIS expression is reduced in pro-inflammatory M1 macrophages and elevated in anti-inflammatory M2 macrophages [51]. Overexpression of PGIS enhances both pro- and anti-inflammatory mediator expression in M1 and M2 macrophages, respectively, whereas PGIS silencing leads to the opposite trends. 6-keto-PGF_1*α*_ is commonly used as a surrogate marker for PGI_2_ levels in most studies as the stable hydrolysis product of PGI_2_ [52]. The observed increase in 6-keto-PGF_1*α*_ levels (*p* < 0.05) following SYB-1 treatment thus reflects enhanced PGI_2_ synthesis and implies the activation of an anti-inflammatory regulatory mechanism within macrophages (Fig. 7A, B). This may serve as a protective response to prevent excessive inflammation, while extracellular PGI_2_ could also exert broader immunomodulatory effects. In contrast, PGF_2*α*_—a pro-inflammatory prostaglandin synthesized from PGH₂ via an alternative enzymatic pathway—signals through the F-prostanoid (FP) receptor to modulate angiogenesis and the expression of inflammatory mediators [53]. However, FP receptor expression is absent in primary immune organs such as the spleen and thymus and has not been reported in immune cell populations, suggesting a limited role for PGF_2*α*_–FP signaling in immune regulation.

As shown in Fig. 7A, B, the upregulation of 6-keto-PGF_1*α*_ and PGF_2*α*_ highlights its role in enhancing prostaglandin metabolism in macrophages, which may be associated with the upregulation of COX-2, PGIS, and PGES. These findings suggest a dual regulatory effect of SYB-1 on both pro- and anti-inflammatory pathways within the prostaglandin network, providing insights into its potential immunomodulatory properties.

(2) The CYP450 pathway.

The CYP450 enzyme family catalyzes AA synthesis in two directions, epoxyeicosatrienoic acids (EETs) (CYP2C and CYP2J as main enzymes) or hydroxyeicosatetraenoic acids (HETEs) (CYP4A and CYP4F as main enzymes) [54]. The former is involved in the inhibition of inflammation, and the latter in the opposite pro-inflammatory direction. It has been extensively demonstrated that 11,12-EET has the best inhibitory effect on inflammation among the four EETs (5,6-, 8,9-, 11,12-, 14,15-EETs) and that 11,12-EET content is reduced in LPS-induced THP-1 macrophages [55, 56]. Tang et al. found depletion of CYP2J2 decreased 11,12-EET level, enhanced inflammatory factor levels, and inhibited M2 macrophage polarization, which were reversed by CYP2J2 overexpression in LPS-treated cells [55]. The above study clarified that 11,12-EET was regulated by CYP2J2, and was negatively correlated with inflammatory response. In addition, the soluble epoxide hydrolase (sEH) can regulate EETs levels, and EETs are further hydrolyzed to the inactive dihydroxyeicosatrienoic acids (DHETs) by sEH. Accumulation of EETs happens in cells from sEH^−/−^ mice and repolarization of classically activated (M1) macrophages into an alternatively activated (M2) [57]. Further, 11,12-EET inhibition of inflammation is associated with downregulation of the NF-κB pathway, which was found in a variety of cells, including macrophages [58, 59]. Indeed, 11,12-EET potently inhibits phosphorylation of IκB-*α* through inhibition of IκB-kinase (IKK) and inactivates NF-κB [60].

In our present study, three types of EET were identified in metabolomics, including 8,9-EET, 11,12-EET, and 14,15-EET. Only 11,12-EET was significantly downregulated (*p* < 0.05) (Fig. 7C–E). Both the downregulation of CYP2J2 and the upregulation of sEH are likely to have caused this change. It is noteworthy that not only does the CYP2 catalyze the production of 11,12-EET, but so does the CYP4A enzyme. However, the production of other EETs is only catalyzed by the CYP2 enzyme [61]. Moreover, CYP4A is involved in the production of HETE with pro-inflammatory effects, so we cannot exclude a regulatory role of SYB-1 on CYP4A, although the metabolomics did not contain results for HETEs.

(3) The anandamide pathway.

The anandamide pathway, which requires the presence of high levels of AA and ethanolamine, produces endogenous cannabinoids such as arachidonoyl ethanolamide (AEA) [62]. Docosatetraenoylethanolamine (DEA), which is structurally similar to AEA, can also be synthesized from AA and is a bioactive endocannabinoid [63]. Due to limited relevant studies, we only know that DEA is generated from AA to produce docosatetraenoic acid, which is further metabolized [64, 65]. Although it is not clear whether DEA is similarly generated via anandamide pathway, the similar structures do not exclude that they would be synthesized via a similar pathway.

In our study, the significant down-regulation of DEA (p > 0.01) may be due to the role of a shift in AA metabolism towards other pathways, such as the COX pathway, in response to SYB-1 intervention (Fig. 7F).

The aforementioned three changes across the AA metabolism indicate that SYB-1 treatment exerts a regulatory effect on macrophage AA metabolism. Specifically, this effect appears to involve the activation of the COX pathway and the potential suppression of both the CYP450 and Anandamide pathways. Given the intimate link between AA metabolism and broader lipid metabolic networks, we sought to identify key molecular targets underlying this regulation. To this end, we constructed a DAMs-gene interaction network associated with the “Fatty Acyls” and “Steroids and steroid derivatives” subclasses by MetaboAnalyst v6.0 (https://www.metaboanalyst.ca/). Using a betweenness centrality threshold of > 1, the resulting network comprised five differential metabolite nodes and twelve gene nodes (Fig. 7G). Among these, the metabolites with the highest degree of connectivity were dodecanoic acid, 11,12-EET, and 6-keto-PGF_1*α*_, suggesting their central roles in the regulatory network. The gene nodes included several members of the CYP450 enzyme family—CYP1B1, CYP3A5, CYP4A11, and CYP4A22—as well as PTGS2, which encodes COX-2. These genes may serve as critical targets through which SYB-1 modulates AA metabolism in macrophages, potentially contributing to its observed immunoregulatory effects.

Glycoproteins can be recognized by pattern recognition receptors (PRRs) on the macrophage surface via their specific glycan chains or protein active sites, including toll-like receptors (TLRs), C-type lectin receptors (CLRs), and DC-SIGN [66]. Engagement of these receptors typically triggers MyD88-dependent activation of NF-κB, as well as phosphorylation of MAPK family members (ERK, p38, and JNK) and the PI3K/Akt pathway, culminating in transcriptional upregulation of proinflammatory genes such as COX-2 [66]. The research by Guo et al. [67] found that glycoproteins from edible fungus *Craterellus cornucopioides* directly activated TLR4, inducing the expression of cytokines as well as activating the cascade of immunoregulatory pathway, while TLR2 also exerts a part of the activation. Zhao et al. [68] reported that *Cirsii Herba* glycoprotein promotes macrophage M1 polarization through MAPK and NF-κB signaling pathways via TLR4.

It is possible that SYB-1 also activates NF-κB or MAPK pathways via such PRRs. In particular, activation of the MAPK pathway may further activate downstream ERK, then the arachidonic acid synthases —PLA_2_ [69]. Consequently, this provides the basis for the synthesis of arachidonic acid to other metabolites, showing the results of regulation on prostaglandins and EETs by SYB-1. However, validation of these deductions requires more probing. Future studies should employ receptor blockade or gene knockdown approaches (e.g., knockdown or inhibition of TLR2/4 and Dectin-1) and assess the effects of SYB-1 on immune signaling pathways and metabolite levels.

## Conclusions

In conclusion, a glycoprotein (SYB-1) with an average molecular weight of 9.423 kDa was purified from *S. biaculeatus*. The total carbohydrate content was 8.46%, composed of mannose, glucose, and galactose with molar ratios of 1.00: 1.34: 2.09. Glycine (19.68 g/100 g), glutamic acid (10.64 g/100 g), alanine (9.36 g/100 g), proline (8.90 g/100 g), and aspartic acid (8.51 g/100 g) were the main amino acids. The total amino acid content determined by amino acid analysis was used as a reference for protein content (88.81%). There may be O-GalNAc and O-GlcNAc glycosylation sites in SYB-1. Linkage analysis of SYB-1 suggested that O-glycosidic bonds were involved in binding between protein and carbohydrate. Furthermore, it was shown that SYB and SYB-1 have significant immunostimulating properties by enhancing the cell viability and promoting the production of NO and cytokines (TNF-*α* and IL-6) in the macrophage RAW264.7. SYB-1 showed enhanced NO and cytokines expression of TNF-*α* and IL-6 compared with the crude SYB. Metabolomics revealed the association of SYB-1 and AA metabolism, including the upregulation of 6-keto-PGF_1*α*_ and PGF_2*α*_, and the downregulation of 11,12-EET and DEA. The CYP450 enzyme family members (CYP1B1, CYP3A5, CYP4A11, and CYP4A22) and PTGS2 may serve as critical targets for the regulatory role of SYB-1. Thus, *S. Biaculeatus* glycoprotein can be considered a nutritional supplement with a potential immunostimulatory property.

## Supplementary Information

Supplementary material 1.

## Data Availability

The data that support the findings of this study are available from the corresponding author upon reasonable request.
