# Supplementary figures and images for: Preparation, physicochemical properties, and immunomodulatory activity of glycoproteins from Syngnathoides biaculeatus
Source: Nat Prod Bioprospect. 2025 Dec 1;15(1):66. doi: 10.1007/s13659-025-00551-6 (PMC12665643; doi:10.1007/s13659-025-00551-6)

**Supplementary Material**

**Figures**


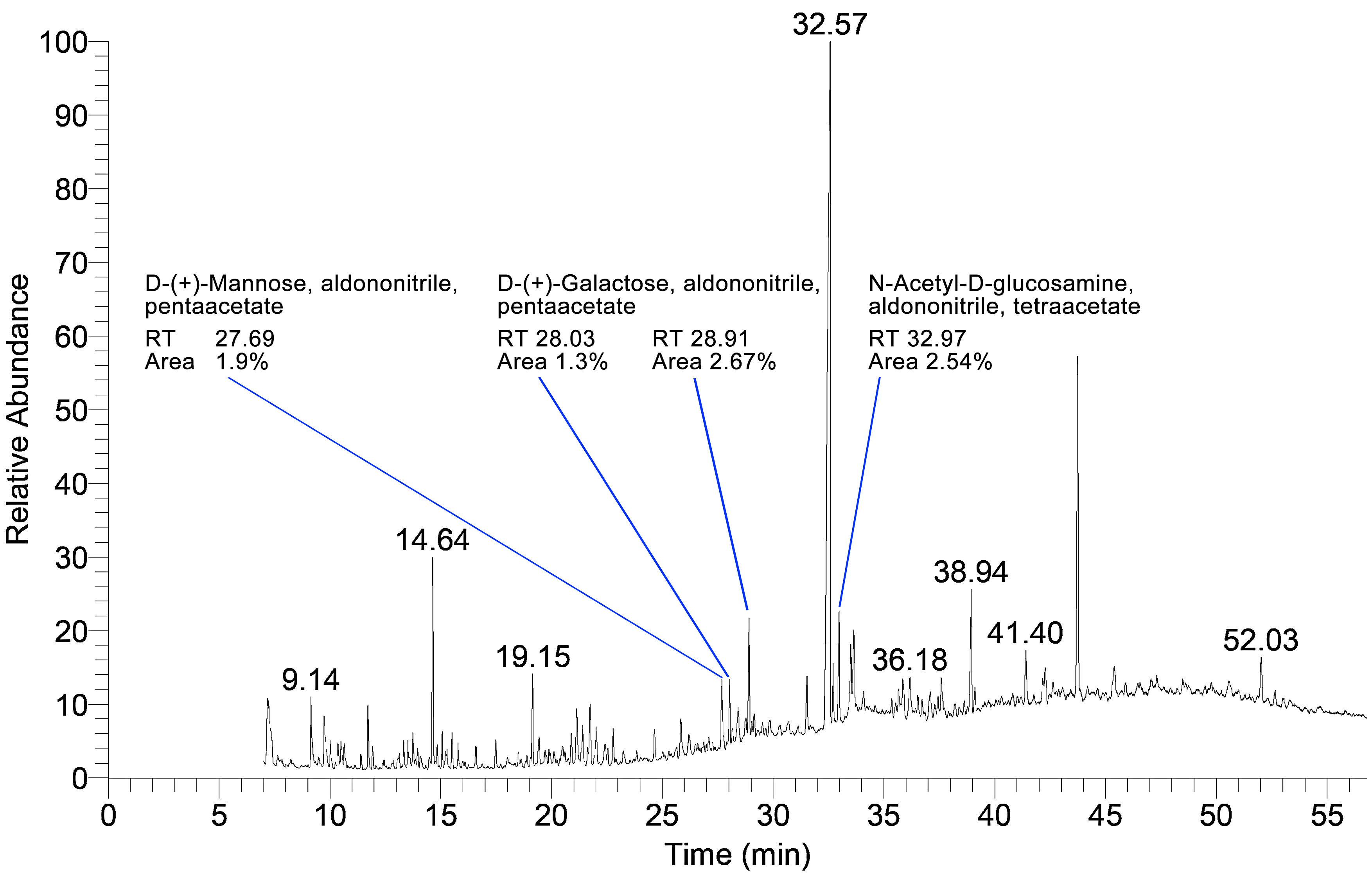


**Figure S1.** GC-MS chromatogram obtained from SYB-1 hydrolysate.

Supplement: Supplementary file 1 — Supplementary material 1. [file 13659_2025_551_MOESM1_ESM.doc]
